# Insight into the evolution of breast cancer driven by genetic alterations

**DOI:** 10.20892/j.issn.2095-3941.2023.0454

**Published:** 2024-02-05

**Authors:** Canbin Fang, Xueqi Fan, Wanling Lin, Guojun Zhang

**Affiliations:** 1 Department of Anatomical and Cellular Pathology, Prince of Wales Hospital, The Chinese University of Hong Kong, Hong Kong SAR 999077, China; 2 Department of Breast Surgery, Yunnan Cancer Hospital and Cancer Institute, The Third Affiliated Hospital of Kunming Medical University, Kunming 650000, China; 3 Department of Breast Surgery, Xiang’an Hospital of Xiamen University, Fujian Key Laboratory of Precision Diagnosis and Treatment in Breast Cancer, Xiamen Key Laboratory of Endocrine-Related Cancer Precision Medicine, Xiamen 361000, China

Breast cancer originates primarily from the epithelial cells of the mammary gland. Repeated mammary gland expansion and degeneration are accompanied by an increased risk of genetic alterations in the breast^1^. These mutations in breast epithelial cells dynamically occur in response to pregnancy, labor and delivery, breastfeeding, and the menstrual cycle, with a decline in mutation rates after menopause, which may be related to a decrease in estrogen levels. The breast epithelial cell mutations are also consistent with epidemiologic observations^2^.

Exposure to internal or external mutagens, flawed DNA sustenance, replication errors, and abnormal DNA editing are responsible for generating multiple mutational processes. In contrast to “passenger” mutations, which are unrelated to the formation of cancer, “driver” mutations are those that provide a cell proliferative advantage and promote the development of a tumor clone. Genomic changes evolve dynamically and continuously throughout the lifespan, and mutations manifest late yet give rise to extensive genomic variety^3^.

With the emergence of high-throughput sequencing technologies, an increasing number of sequence variants have been identified, including single nucleotide polymorphisms (SNPs) and missense or nonsense mutations, which enables the elucidation of disease susceptibility. In addition, significant attention has been directed towards genetic changes associated with microsatellite instability and copy number alterations in oncology involving the deletion, insertion, inversion, and duplication of DNA fragments. Another prominent epigenetic modification is DNA methylation. Notably, The Cancer Genome Atlas Network reported a hyper-methylated phenotype in the luminal B subtype, while the HER2-positive subtype exhibits only a modest association with DNA methylation. Moreover, the loss of 5q and gain of 10p were shown to be correlated with basal-like cancers, while the gain of 1q and/or loss of 16q were highly associated with luminal tumors (**Table 1**)^4^. Baslan et al.^5^ provided a comprehensive overview of copy number variation heterogeneity in breast tumor.

**Table 1:** Genomic, transcriptomic and proteomic features of different breast cancer subtypes^4^ (percentages are based on 466 tumors overlap list)

Subtypes	Luminal A	Luminal B	Basal-like	HER2E
ER+/HER2− (%)	87	82	10	20
HER2 (%)	7	15	2	68
TNBCs (%)	2	1	80	9
TP53 pathway	TP53 mut (12%); Gain of MDM2 (14%)	TP53 mut (32%); Gain of MDM2 (31%)	TP53 mut (84%); Gain of MDM2 (14%)	TP53 mut (75%); Gain of MDM2 (30%)
PIK3CA/PTEN pathway	PIK3CA mut (49%); PTEN mut/loss (13%); INPP4B loss (9%)	PIK3CA mut (32%); PTEN mut/loss (24%); INPP4B loss (16%)	PIK3CA mut (7%); PTEN mut/loss (35%); INPP4B loss (30%)	PIK3CA mut (42%); PTEN mut/loss (19%); INPP4B loss (30%)
RB1 pathway	Cyclin D1 amp (29%); CDK4 gain (14%); Low expression of CDKN2C; High expression of RB1	Cyclin D1 amp (58%); CDK4 gain (25%)	RB1 mut/loss (20%); Cyclin E1 amp (9%); High expression of CDKN2A; Low expression of RB1	Cyclin D1 amp (38%); CDK4 gain (24%)
mRNA expression	High ER cluster; Low proliferation	Lower ER cluster; High proliferation	Basal signature; High proliferation	HER2 amplicon signature; High proliferation
Copy number	Most diploid; Many with quiet genomes; 1q, 8q, 8p11 gain; 8p, 16q loss; 11q13.3 amp (24%)	Most aneuploid; Many with focal amp; 1q, 8q, 8p11 gain; 8p, 16q loss; 11q13.3 amp (51%); 8p11.23 amp (28%)	Most aneuploid; High genomic instability; 1q, 10p gain; 8p, 5q loss; MYC focal gain (40%)	Most aneuploid; High genomic instability; 1q, 8q gain; 8p loss; 17q12 focal ERRB2 amp (71%)
DNA mutations	PIK3CA (49%); TP53 (12%); GATA3 (14%); MAP3K1 (14%)	TP53 (32%); PIK3C (32%); MAP3K1 (5%)	TP53 (84%); PIK3CA (7%)	TP53 (75%); PIK3CA (42%); PIK3R1 (8%)
DNA methylation	-	Hypermethylated Phenotype for subset	Hypomethylated	-
Protein expression	High oestrogen signalling; High MYB; RPPA reactive subtypes	Less oestrogen signalling; High FOXM1 and MYC; RPPA reactive subtypes	High expression of DNA repair proteins, PTEN and INPP4B loss signature (pAKT)	High protein and phospho-protein expression of EGFR and HER2

Amp, amplification; mut, mutation.

Recently, Ogawa^2^ reported the development of breast cancer in carriers of the der(1;16) fusion chromosome, which is also a common driver mutation in approximately 20% of breast cancers, including one-third of luminal A breast cancers and two-thirds of invasive lobular breast cancers. Specifically, Ogawa showed that this driver mutation in breast cancer may occur long before diagnosis. The most recent common ancestor (MRCA) of cancerous and non-cancerous clones appeared in the 18.1–34.4-year-old interval^2,3^ (**Figure 1**). This editorial was inspired by this discovery and aimed to provide an up-to-date summary of studies investigating the evolutionary history and genetic alterations in breast cancers.

**Figure 1:**
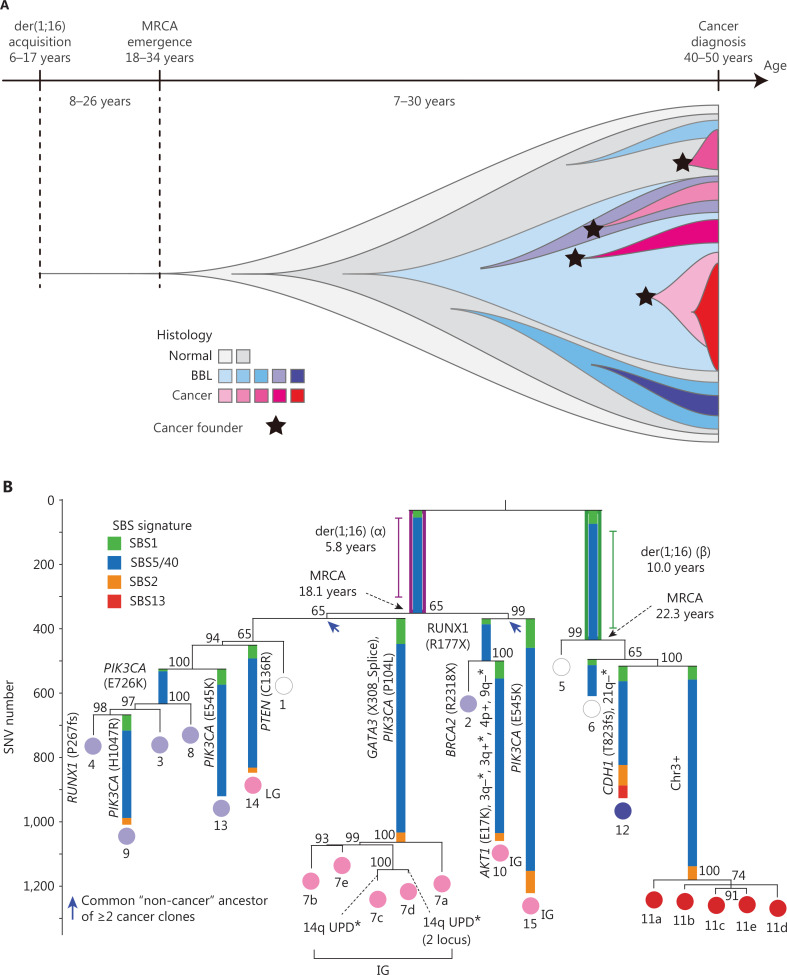
Clonal evolution of breast cancer^2^. (A) Schematic diagram of clonal evolution in premenopausal der(1;16)(+) breast cancer cases is shown with a time course. The colors of each clone depict the histologic features. The black-colored stars indicate multiple cancer founder clones. (B) Phylogenetic tree of a patient with breast cancer who underwent lumpectomy (KU779). The original source of this figure is Ogawa^2^. Adobe Illustrator 2020 (24.1.2 64 bit) was used to generate this figure.

## Genetic alterations and breast cancer evolution

Our understanding of driver and passenger genes in somatic mutations has rapidly brought tumor treatment into the era of precision treatment. In addition, dysregulation of crucial signaling networks that regulate cell survival, proliferation, and/or differentiation pathways tends to be the outcome of multiple somatic genetic changes in breast cancer. To date, numerous gene mutations related to breast cancer have been discovered through whole genome sequencing (WGS), among which PI3KCA, TP53, and MAP3K1 have been shown to be highly mutated. In addition, PIK3CA, TP53, and PTEN appear to be clonally dominant, all of which are involved in the early stages of breast cancer development, suggesting a role in the evolution of breast cancer^6^.

In the past several decades, a number of drugs targeting such mutations have been developed for breast cancer, such as those targeting human epidermal growth factor receptor 2 (HER2), cyclin-dependent kinase (CDK) 4/6 inhibitors, poly ADP-ribose polymerase (PARP) inhibitors targeting BRCA1/2 mutations, and immune checkpoint inhibitors. According to the NCCN guideline recommendations for breast cancer, the following tests should be selectively carried out in breast cancer patients at different stages: 21 genes/70 genes/50 genes/12 genes; HER2; BRCA1/2; PIK3CA; NTRK fusion; PD-L1 status; MSI-H/dMMR; and tumor mutation burden (TMB). Comprehensive genetic testing can bring tangible clinical benefits to breast cancer patients, such as guiding targeted therapy, predicting resistance, assessing genetic risk, and assisting surgical decisions.

Despite significant advances in comprehensive therapeutic strategies and early diagnosis, drug resistance and tumor recurrence remain major challenges in breast cancer management. These challenges can be attributed to the extensive inter- and intra-tumor heterogeneity acquired during carcinogenesis and the evolution of breast cancer. Therefore, understanding the evolutionary history of breast cancer is also crucial for unraveling the complexities of tumor heterogeneity and improving breast cancer treatment and prevention strategies.

## Clonal expansion of epithelial cells and heterogeneity of breast cancers

**Figure 2:**
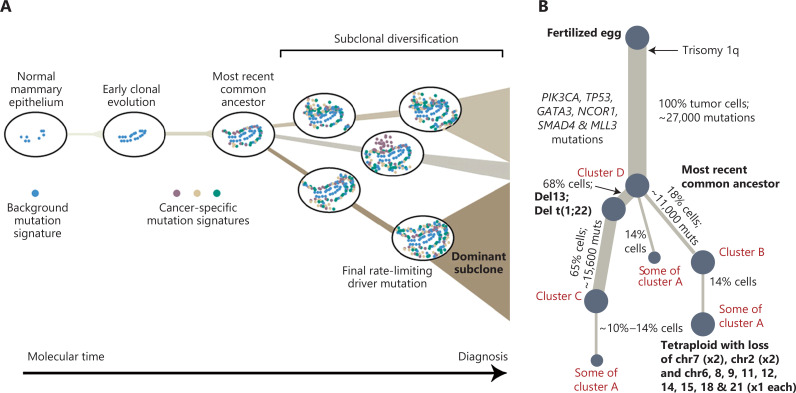
Graphical abstract of breast cancer evolution^3^. (A) Cancer evolves dynamically as clonal expansions supersede one another driven by shifting selective pressures, mutational processes, and disrupted cancer genes. Expansion of the dominant subclone to an appreciable mass may therefore represent the final rate-limiting step in breast cancer development, triggering diagnosis. (B) Reconstruction of the phylogenetic tree of a patient carrying various mutations. Thickness of the branches reflects the proportion of tumor cells comprising that lineage. The length of the branches reflects the number of mutations specific to that lineage. The original source of this figure is Campbell^3^. Adobe Illustrator 2020 (24.1.2 64 bit) was used to generate this figure.

Cancer is the result of continuous evolutionary selection of cells undergoing multi-step and multimolecular changes, beginning with normal epithelial cells and progressing to atypical ductal hyperplasia and ductal/lobular carcinoma *in situ*, and ending as invasive ductal/lobular carcinoma with metastasis^7^ (**Figure 2**). Thus, the clonal expansion of epithelial cells might originate from multiple sites and result in inter-tumoral heterogeneity. In addition, the metabolic heterogeneity of breast cancer may be caused by the genetic metabolic profile of normal breast cells, as reported by Mahendralingam et al.^8^, who discovered that certain subtypes of breast cancer preserve the metabolic traits of the presumed cells of origin. There is compelling evidence that luminal A breast cancers are similar to mature luminal cells. More specifically, mature luminal cells in BRCA1 mutation carriers may be the origin of ER^high^ luminal breast cancers. In addition, luminal progenitor cells are the precursors for familial BRCA1-mutated tumors and basal-like cancers^9^.

Analysis of tumor tissue alone often obscures the sequence of early driving events, which are often assigned to long trunks in the phylogenetic tree and fails to trace the evolutionary history of tumor and non-tumor clones. Interestingly, Nishimura et al.^2^ reconstructed the phylogenetic tree of tumors and non-tumor clones by performing WGS on normal epithelial tissues, non-cancerous lesions, and breast tumor tissues. The evolution of breast cancer and precursor lesions was successfully tracked from obtaining initial driver alterations to the development of clinically diagnosed disease. Further studies showed that there are multiple competing clones within the same lesion^10^. In addition, single-cell analysis also revealed significant heterogeneity of epithelial, immune, and mesenchymal phenotypes presented in each tumor, which differs from previous bulk studies that relied on an *a priori* molecular subtype^11^.

## Identification of genetic alterations of intrinsic subtypes

The large somatic tumor mutation dataset shows that breast cancer is highly mutated, but there are few related high-frequency mutations, suggesting that there are significant differences between breast cancer molecular subtypes and there is no single breast cancer pathogenic molecule.

The mutational spectrum of luminal/ER+ breast cancers is distinct and heterogeneous, including the frequent mutation of MAP2K4 and MAP3K1, the high mutation frequency of PIK3CA, the differentially inactivated TP53 pathway, and high ESR1, FOXA1, MYB, GATA3, and XBP1 mRNA and protein expression.

An HER2/phosphorylated HER2/EGFR/phosphorylated EGFR signature was shown to be dominant in HER2-amplified tumors^4^. It is interesting to note that HER2+ and HER2− areas have distinct heterogeneous distributions of drivers and frequent alternative gene copy number alterations (CNAs) in the HER2-enriched subtype, suggesting that HER2− regions have unique driver events. Furthermore, Berrino et al.^12^ debated that HER2-low breast cancer (HLBC) is a distinct entity. HLBCs harbor unique genomic features by genomic and transcriptomic analyses when compared to HER2-positive BCs. The ATM mutations, loss of RB1, inactivation of BRCA1, and amplification of cyclin E1 are top-scoring modules in basal-like tumors^4^ (**Table 1**). Minussi and colleagues^13^ concluded that TNBCs continue to preserve a subcloned diversity and evolve chromosome aberrations during primary tumor growth. According to convincing evidence generated by Jacobson et al.^14^, BRCA1/2-defect status has significant heterogeneity with respect to homologous recombination deficiency. Notably, it was discovered that poorer outcomes are associated with more intratumoral heterogeneity, including copy number and mutation patterns across multiple subtypes of breast cancer^15^.

## Role of driver mutations in breast cancers

### der(1;16) chromosomal translocation

The chromosomal translocation, der(1;16), involves fusion of chromosomes 1 and 16. Fluorescence *in situ* hybridization (FISH) has revealed that der(1;16) is a fusion of 1q12 and 16cen or 16q11.2. Notably, der(1;16) is frequently detected in low-grade (grade 1) papillary carcinoma but not benign papillomas. Therefore, der(1;16) serves as a potential indicator of low-grade or well-differentiated breast carcinoma^16^; however, the mechanism underlying der(1;16) formation and its involvement in the activation or inactivation of specific tumor-associated genes during mammary carcinogenesis is unclear. A recent study conducted by Nishimura et al.^2^ shed light on the evolutionary history of breast cancers harboring der(1;16). Nishimura et al.^2^ found that the derivative chromosome in der(1;16)(+) cancers is acquired during the transition from early puberty-to-late adolescence. Subsequently, a common ancestor emerged early in the 4^th^ decade of life from which cancer and non-cancer clones evolved. Over the following years, these clones replaced the pre-existing mammary epithelium and occupied a significant area within the premenopausal breast tissues by the time of cancer diagnosis. The evolution of multiple independent cancer founders from non-cancer ancestors was prevalent, thereby contributing to intratumoral heterogeneity.

### AKT1 mutation

AKT1, a serine/threonine protein kinase, has a critical role in cell survival, growth, proliferation, metabolism, and angiogenesis. The AKT1 protein is a key component of the PI3K/AKT signaling pathway, which is frequently dysregulated in cancer. The most common AKT1 driver mutation in breast cancer is the E17K (G49A) mutation. The pleckstrin homology (PH) domain of AKT1 is where this mutation arises, leading to a substitution of glutamic acid (E) with lysine (K) at position 17^17^. Consequently, the E17K mutation induces a conformational change in the PH domain, which causes constitutive membrane localization and activation of AKT1 independent of upstream signals.

These mutations can occur at different stages of tumor development and contribute to the progression and aggressiveness of the disease. An AKT mutation can confer a growth advantage to a subset of cancer cells in the early stages of breast cancer. As a result, the mutated cells can outcompete neighboring cells and become the dominant population within the tumor. Over time, the AKT-mutated cells continue to evolve and acquire additional genetic alterations. These alterations further enhance the invasive properties, allowing the AKT-mutated cells to invade surrounding tissues and metastasize to distant sites^18^.

### PIK3CA mutation

One of the most typical mutations in breast cancer is PIK3CA, a gene that codes for the catalytic subunit of the PI3K enzyme. Approximately 30%–40% of breast cancer cases harbor PIK3CA mutations. These mutations predominantly occur as hotspot mutations in 2 specific regions of the gene: exon 9 (helical domain); and exon 20 (kinase domain). E545K in exon 9 and H1047R in exon 20 are the most frequent hotspot mutations. An activating PIK3CA mutation promotes cell survival, growth, proliferation, and metabolic reprogramming, contributing to tumor initiation and progression. An activating PIK3CA mutation also inhibits apoptosis, stimulates cell cycle progression, and facilitates angiogenesis and metastasis^19^.

## Discussion and perspectives

Assessment of clones evolution with typical cancer genetic mutations in normal tissues has been rendered possible by sequencing procedures, but the additional driver mutations that occur during this progression and the order in which the driver mutations appear have not been fully elucidated. Using phylogenetic analyses of laser capture microdissected samples from both cancerous breast lesions and multifocal non-cancer proliferative lesions, Nishimura and colleagues^2^ detailed the evolutionary history of breast cancer with the constructed phylogenetic trees and the progeny occupying a large area of the premenopausal breast (**Figure 1B**).

Moreover, the Nishimura et al.^2^ study demonstrated the most recent common ancestor commonly harbors der(1;16), a driver alteration of breast cancers. Assessment of the timing of der(1;16) acquisition, as estimated from the mutation rate measured in normal epithelial cells, indicated the derivative chromosome was acquired during early puberty-to-late adolescence, with the emergence of the common ancestor early in the 4^th^ decade of life. Further characterization indicated that the expansion of der(1;16)(+) clones not only is explained by physiologic development but suggests a driver role for der(1;16). Similar evolutionary patterns were also observed with the AKT1 driver mutation. Additionally, multiple cancer clones have been shown to commonly evolve from non-cancer ancestors and a lack of correlation has been detected between histology and the number of driver events, suggesting that epigenetic or microenvironmental features have a role in cancer development.

It is interesting to note that cancer clones frequently developed multifocally from clonally similar but “non-cancer” progenitors. During the formation of cancer, a branching pattern of evolution involving several cancer founders from within a non-cancer population occurs more frequently than predicted. This implies that regionally defined microenvironments and/or epigenetic modifications have a partial role in the genesis of cancer^20^.

Importantly, a significantly reduced mutation rate after menopause might be associated with reduced cell turnover due to reduced estrogen levels. In contrast, the accumulation of mutations is enhanced by pregnancy and delivery, during which estrogens are elevated while menstrual cycles are spared. This finding suggests that newly recruited “dormant” stem cells, in which the SNV burden has been spared, could reconstruct the mammary epithelium following the effacement of significantly proliferative mammary glands after delivery or breastfeeding.

In summary, this work provides insight into breast cancer evolution spanning initial driver alteration acquisition by revealing the timing and order of these early driver events in clinically diagnosable disease development. Therefore, mechanisms of mutation in the mammary epithelium and the full course of breast cancer may highlight the role of driver mutations, such as the importance of der(1;16) in the primary subset of luminal A breast cancer. Future studies focusing on the evolutionary history contributes to the emergence of novel methods for early prediction, detection, and potential prevention of breast cancer.
